# Maternal Prenatal External Locus of Control and Reduced Mathematical and Science Abilities in Their Offspring: A Longitudinal Birth Cohort Study

**DOI:** 10.3389/fpsyg.2019.00194

**Published:** 2019-02-08

**Authors:** Jean Golding, Steven Gregory, Genette Ellis, Terezinha Nunes, Peter Bryant, Yasmin Iles-Caven, Stephen Nowicki

**Affiliations:** ^1^Centre for Academic Child Health, Population Health Sciences, Bristol Medical School, University of Bristol, Bristol, United Kingdom; ^2^Department of Education, University of Oxford, Oxford, United Kingdom; ^3^Department of Psychology, Emory University, Atlanta, GA, United States

**Keywords:** ALSPAC, longitudinal cohort, maternal locus of control, numeracy, science, mathematics

## Abstract

A personality scale that identifies individuals’ general attitude to what happens to them as largely a matter of luck or fate or of powerful others (externality) or whether they feel they can influence the consequences (internality) is known as locus of control (LOC). A continuous scale can distinguish those who are more external from those who are more internal. Lower scholastic achievement is associated with externality and higher achievement with internality, but little is known about the association of parental LOC on children’s academic performance. Data collected within the Avon Longitudinal Study of Parents and Children (ALSPAC) are analyzed to assess associations between mothers’ LOC orientation, measured during pregnancy, and their children’s abilities in mathematics and science reasoning. We found that maternal external LOC is associated with lower scores for her child assessed by tests measuring mental arithmetic as well as understanding of mathematical and scientific concepts. Additionally, we determined the extent to which three separate sets of factors previously found to positively influence the developing child’s ability mediate these findings: (a) perinatal and infant exposures, such as prenatal smoking, binge drinking, consumption of oily fish, and postnatal breast feeding; (b) parenting attitudes and strategies; and (c) the interface of the parents with their child’s school. The three factors identify at least 50% of the mechanism by which maternal externality is associated with poor academic outcomes in her child and may be candidates for further investigation as possible intervention targets.

## Introduction

As Meadows et al. (2008) have stated: ‘Improving numeracy learning has been a dominant educational theme throughout the development of statutory education over the last 100 years or so.’ At an individual level, numeracy has a profound effect on overall quality of life, self-identity and the capacity to function in an ever more complex world; and at a macro level, the success of society depends on a well-educated, numerate and adaptable workforce. However, with the recent advent of the digital age and the need for skilful use of information communications technology, the importance of children becoming proficient with numeracy skills is becoming ever more pressing’ (Meadows et al., 2008). Almost equally important nowadays is the parallel development of scientific understanding.

In this paper we attempt to determine a pathway to increased ability in maths and science by assessing ways in which the mother’s locus of control (LOC) may influence the child’s outcome. Rotter (1966) first introduced the concept of LOC, which he defined as follows: ‘Internal versus external control refers to the degree to which persons expect that a reinforcement or an outcome of their behavior is contingent on their own behavior or personal characteristics versus the degree to which persons expect that the reinforcement or outcome is a function of chance, luck, fate, is under the control of powerful others, or is simply unpredictable. Such expectancies may generalize along a gradient based on the degree of semantic similarity of the situational cues.’

The introduction of the concept of LOC was greeted positively by both the psychological and educational communities. One of the reasons it has maintained its popularity, as reflected in the well over 17,000 studies that have used it (Nowicki and Duke, 2016), is because of its association with academic achievement. In the same year that Rotter introduced the concept, Coleman et al. (1966) published the results of a national study of half a million students showing their own LOC to be one of the best predictors of their academic achievement. Since that time the LOC, academic achievement association has been examined in many ways, but most often by simply correlating students’ LOC orientation with some measure of academic achievement such as grades or standardized tests. Two major reviews (Findley and Cooper, 1983; Kalechstein and Nowicki, 1997) of the LOC, academic achievement association, separated by nearly a decade and a half, came to almost identical conclusions: internally controlled children of all ages did better academically than their externally controlled peers, even when IQ was controlled for.

While most past studies have concentrated on correlating individuals’ LOC with some current measure of academic achievement, others have attempted to use LOC to predict future outcomes tied to academic achievement. One example was a study completed by Flouri (2006) who used data from the British Cohort Study (BCS70), a longitudinal study originating in 1970 that included a representative sample of children born in different regions of Great Britain. She found that 10-year-olds’ LOC scores as measured by an Anglicized form of the Nowicki-Strickland Internal External control scale (Nowicki and Strickland, 1973), predicted their educational attainment some 26 years later. Taking account of factors such as parental social class, cognitive ability, socioeconomic disadvantage and maternal educational attainment, analyses of the responses of 1,326 men and 1,578 women revealed that their own internality in childhood, along with their mothers’ greater interest in their children’s achievements, were related to their final educational attainment in both the men and women.

Thus, (1) we know internality is associated with greater academic achievement throughout the lifespan and (2) both mothers’ interest in their children’s academic pursuits, coupled with the children’s internality, predict future academic accomplishment. However, somewhat surprisingly, we know relatively little about the contribution of parents’ own LOC orientation to their children’s future academic achievement. From what we know about the LOC construct, however, it is likely that parental LOC orientations may affect how they may be able to deal with the everyday challenges of raising their children to be successful academically.

Some help in predicting how the LOC orientation of parents may be associated with the upbringing of their children comes from a summary of study results. Nowicki (2016b) suggests, compared to internals, externals generally: (1) show less persistence in attempting to solve problems, (2) feel less responsibility for their behavior, (3) are rarely relentless in the pursuit of information, (4) cannot tolerate long delays of gratification, and (5) show less resistance to being coerced; characteristics that may not be helpful in attempting to parent successfully.

Based on empirical findings and Rotter’s assumptions regarding LOC, we predicted that children whose mothers are more external will: (a) have poorer educational achievements; (b) have relationships between maternal LOC and the child’s mathematical and science abilities that will be mediated through maternal lifestyle, parenting behaviors and attitudes to education.

## Materials and Methods

### The Study Sample

This study takes advantage of the data collected as part of the Avon Longitudinal Study of Parents and Children (ALSPAC) a pre-birth cohort which was designed to determine the environmental and genetic factors that are associated with health and development of the study offspring (Golding and ALSPAC Study Team, 2004; Boyd et al., 2013; Fraser et al., 2013).

ALSPAC recruited 14,541 pregnant women resident in Avon, UK with expected dates of delivery 1st April 1991 to 31st December 1992 (an estimated 80% of the eligible population). 14,541 is the initial number of pregnancies for which mothers enrolled in the ALSPAC study had either returned at least one questionnaire or attended a hands-on assessment (“Children in Focus” clinic) by the 19th July 1999. Of these initial pregnancies, there was a total of 14,676 fetuses, resulting in 14,062 live births and 13,988 children who were alive at 1 year of age. Data were collected at various time-points using self-completion questionnaires, biological samples, hands-on measurements, and linkage to other data sets. Please note that the study website contains details of all the data that are available through a fully searchable data dictionary and variable search tool: http://www.bristol.ac.uk/alspac/researchers/our-data/

Ethical approval for the study was obtained from the ALSPAC Ethics and Law Committee and the Local Research Ethics Committees (Birmingham, 2018). Detailed information on the ways in which confidentiality of the cohort is maintained may be found on the study website: http://www.bristol.ac.uk/alspac/researchers/research-ethics/

The study design included a concerted effort before the child’s birth to obtain from the parents details of their personalities, moods and attitudes, including a measure of their LOC. This involved the pregnant women completing four questionnaires during the pregnancy, one of which contained the LOC scale; in parallel they were sent two questionnaires for their partners to complete, one of which included the identical LOC scale.

### The LOC Measure

The LOC measure used in the present study is a shortened version of the adult version of the Nowicki-Strickland Internal-External locus of control scale (ANSIE) comprising 40 items in a yes/no format to assess perceived control (Nowicki and Duke, 1974). This measure was chosen over other scales more specifically related to perceived control over health, as it was considered that this more generalized scale would relate to other factors in addition to health outcomes. Construct validity for the scale has been found in the results of over a 1000 studies (Nowicki, 2016a). The version used in the present study comprises 12 of the original 40 items which were chosen after factor analysis of the ANSIE administered as a pilot to 135 mothers. The 12 questions loaded onto a single factor of general LOC. The 12 questions used are shown elsewhere (Golding et al., 2017b). From the responses a ‘LOC score’ was derived, the higher the score the more external the LOC. The scores ranged from 0 to 12. For this study, external LOC was defined as having a score greater than the median. This cut-off identified 45.2% of the women and 46.6% of the men as externally controlled (ELOC).

Test–retest reliability was assessed in this study by comparing the results 6 years later. The correlations were strong for both men and women (*r* > 0.50).

### Outcome Measures

#### Mathematical Reasoning

These tests were devised by Nunes and Bryant for the ALSPAC study. Their aim was to assess children’s understanding and use of quantitative relations to solve mathematical problems.

Two different Mathematical Reasoning tasks were designed. The one containing 17 items, was given to school-children inYear 4 (*N* = 5275, mean age 8 years 9 months). Three types of item were included in the Year 4 Mathematics Reasoning Task: additive reasoning items about quantities, additive reasoning items about relations, and multiplicative reasoning items about quantities. The assessments used in Years 6 and 8 included six types of item: additive reasoning items about quantities; additive reasoning items about relations; multiplicative reasoning items about quantities; multiplicative reasoning items involving relations (i.e., proportions); items about spatial reasoning and items about fractional quantities.

The aim of each task was to assess children’s reasoning about quantities and the relations between quantities in mathematical problems independently of their computation skills. None of the items in these tests contained difficult calculations; the children had to reflect on the relations between quantities in each item in order to decide how to solve the problem. All the items were presented with the support of drawings; the children could use counting to solve many of the problems if they did not know the number facts that might be used in the solution. All the problems were presented orally by the teachers to avoid an undue influence of reading difficulties on the children’s performance (Nunes et al., 2009).

Analyses of their internal consistency using Cronbach’s α showed that on all three occasions the mathematics reasoning tasks had good levels of inter-item reliability: 0.74 at Year 4 (*N* = 5275), 0.89 at Year 6 (*N* = 7881) and 0.91 at Year 8 (*N* = 2755). This high internal consistency justifies the addition of all the items into a single score.

#### Mental Arithmetic

Mental Arithmetic was measured as part of the WISC (Wechsler et al., 1992) intelligence test administered at 8 years by trained psychologists. The raw scores at age 8 years were measured using alternate questions as for the WISC test in general (Golding et al., 2017a). The scores were approximately normally distributed. As reported by Nunes et al. (2012): *“the split-half reliability for 8-year-olds is 0.78 (Wechsler, 1992, p. 60); and the average correlation with the Wide Range Achievement Test Arithmetic Score is 0.62 (Wechsler, 1992, p. 76), which makes this a valid and economical assessment of children’s arithmetic knowledge, thus, suited for large-scale studies such as this.”*

#### Scientific Understanding

This used a test designed to examine the child’s understanding of the concepts used in science. It was designed by Terezhina Nunes (TZ) and Peter Bryant (PB) to be administered in school (Bryant et al., 2015). This was undertaken in School Year 6 at the same time as the year 6 maths test (see above). Cronbach’s alpha for this task with the sample of 4,046 children who participated in the study was 0.721, and Bryant et al. (2015) showed strong correlations with the SATS 2 scores.

### Variables Controlled For

All analyses included the sex of the child together with the mother’s parity (i.e., the number of previous pregnancies resulting in a live- or still-birth) at the time of birth of the study child (0, 1+). In addition, the following analyses were undertaken.

#### Prenatal Chemical Exposures

The following variables were included since there is considerable evidence to implicate them in neurocognitive development: (i) maternal cigarette smoking mid-pregnancy (yes/no); (ii) maternal binge drinking mid-pregnancy (4 or more units of alcohol on at least 1 day in the past month) (yes/no); (iii) frequency of maternal consumption of oily fish in third trimester (none/any); (iv) duration of breast feeding (none or < 4 weeks/at least 4 weeks).

#### Parenting Strategies in Early Childhood

The factors included were: (i) frequency mother sings to the child at 24 months; (ii) the frequency that the mother reads to the child at 24 months; (iii) a parenting score derived from the frequency with which the mother attempts to teach and interact with the child at 24 months (<33/33+ score ranged from 18 to 40); (iv) frequency with which mother takes the child to a library at 30 months (rarely or never v. at least several times a year); (v) frequency mother takes child to places of interest at 30 months (never vs. at least several times a year); (vi) frequency is allowed objects to build with (once a week or less vs. more than once a week); (vii) exposed to people smoking when aged 3; (viii) has a diet of ‘junk’ food at age 3 (see Feinstein et al., 2008); (ix) mother’s partner reads to the child at age 57 months (often/<often).

#### Variables Based on Fostering Educational Achievements

The following variables were included in this analysis: (i) no. of books owned by the child at age 6 (<20, 20–49, 50+); teacher report of the following: (ii) no. of children excluded from school in year 3 (0,1+); (iii) no. of children receiving free school meals in year 3 (0, 1–14, 15+); (iv) no. of disadvantaged pupils in the school (continuous); (v) frequency child does homework in year 3 (never vs. rest); (vi) teacher report of how supportive the study child’s parents are (very supportive vs. rest).

### Statistical Approach

**FIGURE 1:**
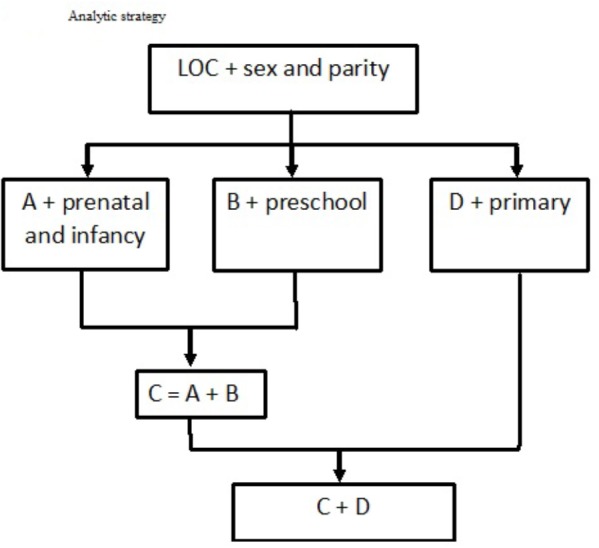
Analytic strategy.

The analyses are designed to determine the relationship between the children’s mean scores on the mathematics and science tests and the mother’s LOC orientation. The basic data use backward stepwise multiple regression adjusted for sex and parity since these are not open to modification once the child is conceived. For each analysis we noted the regression coefficient (b), the measure of variance explained (*R*^2^) and the statistical significance (*P*). The analysis was then repeated but taking account of the prenatal chemical exposures (Model A). A separate analysis allowed for the parenting strategies (Model B), and a further analysis combined factors A and B (Analysis C). The fourth analysis allowed for factors indicating the fostering of the school-age child’s ability (Analysis D). Subsequent analyses enabled all the factors in C and D to be taken into account together. Comparison of the regression coefficients and the amount of variance explained for each model was used to deduce the contribution of the different factors in explaining the ways in which the maternal LOC may have impacted on the child’s scores. The analytic strategy is illustrated in Figure 1.

## Results

### Response Rates by Maternal LOC

With the exception of the mental arithmetic score, which was only obtained by the study if the child was brought to the ALSPAC research clinic, the other tests of mathematics and science depended on the response rates of teachers rather than of the study families. As can be seen from Table 1, for the measures assessed in school, compared with all children, the proportions of responders whose mothers were external varied little, but there was a difference for the mental arithmetic with proportionately fewer children of external mothers.

**Table 1:** Proportion of children answering the different tests according to the locus of control orientation of their mothers in pregnancy.

Test	*N*	Mother external %	Mother internal %
Math Year 4	4623	43.2	56.8
Math Year 6	6926	44.3	55.7
Math Year 8	2453	41.9	58.1
M.A.	6842	38.0	62.0
Science	6951	44.6	55.4
All children	12471	45.2	54.8

*M.A, mental arithmetic.*

The ways in which the basic social and environmental measures varied with the groups of children who completed the different measures is illustrated in Supplementary Table 1. In general there were few differences, although the group of children who completed the mental arithmetic test were less likely to have young mothers, mothers who smoked in mid-pregnancy or mothers with low levels of education.

### The Measures

The basic details of each score are shown in Supplementary Table 2, and the correlations between the different scores are shown in Table 2. There are correlation coefficients ranging from 0.388 to 0.590 for the relationships between the test scores, and from 0.424 to 0.592 for their relationships with the total IQ score. Interestingly the maths scores tended to have relatively low correlations with the science reasoning score (range 0.388–0.480).

**Table 2:** Correlation matrix between the mental arithmetic, the three maths comprehension, the science, and total IQ scores (*n* = 438).

Test	M.A	Math Y4	Math Y6	Math Y8	Science	IQ
M.A	1.00					
Math Y4	0.477	1.000				
Math Y6	0.476	0.518	1.000			
Math Y8	0.397	0.487	0.590	1.000		
Science	0.388	0.401	0.480	0.415	1.000	
IQ	0.592	0.491	0.558	0.525	0.424	1.000

*M.A., mental arithmetic; Y, school year.*

### Initial Analyses by Maternal LOC

Allowing for sex of the child and maternal parity we show in Table 3 that the children of mothers with an external LOC have strong negative relationships with the results of the tests, with results varying from differences of 0.92–2.82 SDs and *P*-values of 10^−22^ to 10^−62^. These results took account of the child’s sex which showed female reductions of between 0.25 and 1.79 SDs for the mathematics reasoning and arithmetic tests, with *P-*values from 0.006 to 10^−27^, but no sex differences for the science reasoning test. Parity, as a proxy for the presence of older siblings, was only independently associated with maths and science reasoning in Year 6; both indicated that having an older sibling was associated with poorer achievement. It is clear from the results of all five tests that maternal external LOC has the largest associations (<-2 SDs) with mathematical reasoning in school years 6 and 8, although all the differences are highly statistically significant.

**Table 3:** Stepwise logistic regression results for sex of child, parity and maternal external locus of control for each of the mental arithmetic, the three maths comprehension, and the science scores.

Test	*N*	Ext LOC		Sex		*R*^2^ %
		β [95%CI]	*P*	β [95%CI]	*P*	
Math Y4	4623	−1.21 [−1.39, −1.03]	3.8 × 10^−40^	−0.25[−0.42, −0.07]	0.006	3.89
Math Y6	6665	−2.82 [−3.15, −2.49]	2.8 × 10^−62^	−1.79[−2.12, −1.47]	7.0 × 10^−27^	5.97^a^
Math Y8	2453	−2.76 [−3.32, −2.20]	7.6 × 10^−22^	−1.44[−1.99, −0.88]	3.5 × 10^−7^	4.68
M.A.	6787	−0.92 [−1.09, −0.76]	3.7 × 10^−27^	−0.36[−0.52, −0.20]	1.4 × 10^−5^	1.98
Science Y6	6689	−0.95 [−1.07, −0.83]	2.1 × 10^−52^	DNE		3.72%^b^

*M.A., mental arithmetic; DNE, did not enter model; Y, school year. ^a^Model also includes parity −0.63 [−0.96, −0.31] P = 1.5 × 10^−4^. ^b^Model also includes parity −0.24 [−0.36, −0.12] P = 9.2 × 10^−5^.*

### Explanation by Features of Pregnancy, Infancy, and the Preschool Period

We examined the extent to which factors relevant to pregnancy and infancy and known to be associated both with the mother’s LOC and the child’s cognitive development, might explain a proportion of the mechanism by which the mother’s externality is associated with a reduction in maths and science abilities (Analyses A). Thus, in Table 4 we show the change in effect size associated with a combination of maternal youth, failure to eat oily fish, prenatal smoking in mid-pregnancy, binge drinking mid-pregnancy and failure to breast feed at or beyond 4 weeks of age. The reduction of maths and science scores attributed to external LOC reduced by about a third, but mental arithmetic by slightly less (27%).

**Table 4:** Reductions in the effect size of maternal external locus of control after taking account of prenatal and infancy factors^a^ as well as sex and parity, for each of the mental arithmetic, the three maths comprehension, and the science scores: results of stepwise regression.

Test	*N*	Adjusted β [95%CI]	Reduction^b^ %	*P*	*R*^2^ %
Math Y4	3987	−0.79 [−0.99, −0.60]	35	5.7 × 10^−15^	6.81
Math Y6	5875	−1.80 [−2.16, −1.44]	38	8.0 × 10^−23^	10.72
Math Y8	2252	−1.84 [−2.42, −1.25]	33	1.1 × 10^−9^	7.69
M.A.	6188	−0.67 [−0.85, −0.49]	27	2.3 × 10^−13^	3.73
Science Y6	5924	−0.64 [−0.77, −0.51]	34	4.1 × 10^−21^	7.37

*^a^Adjusted for maternal age, consumption of oily fish in pregnancy, smoking cigarettes mid pregnancy, binge drinking mid-pregnancy, and breast feeding. ^b^Reduction in the adjusted beta coefficient for external locus of control compared with results in Table 3.*

Independent of Analyses A we assessed the degrees to which the lower scores of children with external mothers were ‘explained’ by the parenting behaviors in the preschool period as defined by visits to library, visits to places of interest, mother sings to child, mother reads to child, parenting score, child allowed objects for building, exposed to environmental tobacco smoke, child’s diet is poor (named ‘junk food diet’). These factors (Analyses B) accounted for 38–58% of the reduced maths and science scores (Table 5).

**Table 5:** Reductions in the effect size of maternal external locus of control after taking account of preschool parenting factors^a^ as well as sex and parity, for each of the mental arithmetic, the three maths comprehension, and the science scores: results of stepwise regression.

Test	*N*	Adjusted β [95%CI]	Reduction^b^ %	*P*	*R*^2^ %
Math Y4	3316	−0.75 [−0.96, −0.54]	38	6.9 x 10^−12^	7.15
Math Y6	4864	−1.51 [−1.90, −1.11]	48	1.1 x 10^−13^	10.14
Math Y8	1606	−1.17 [−1.87, −0.47]	58	0.001	10.31
M.A.	5050	−0.48 [−0.68, −0.28]	49	3.6 x 10^−6^	4.63
Science Y6	4872	−0.55 [−0.70, −0.401	44	5.3 x 10^−13^	7.04

*^a^Adjusted for visits to library, visits to places of interest, mother sings to child, mother reads to child, parenting score, child allowed objects for building, exposed to environmental tobacco smoke, child’s diet is poor (named ‘junk food diet’). ^b^Reduction in the adjusted beta coefficient for external locus of control compared with results in Table 3.*

Offering all features of Analyses A and B to the regression (Analyses C; Table 6) indicated that they explained between 47 and 65% of the reduction in scores of the children of externally oriented mothers. The size of the reduction was greatest for mathematical reasoning in School Year 8, and least for scientific reasoning.

**Table 6:** Reductions in the effect size of maternal external locus of control after taking account of prenatal, infancy and preschool factors^a^ as well as sex and parity, for each of the mental arithmetic, the three maths comprehension, and the science scores: results of stepwise regression.

Test	*N*	Adjusted β [95%CI]	Reduction^b^ %	*P %*	*R*^2^ %
Math Y4	3069	−0.61 [−0.83, −0.38]	50	1.4 × 10^−7^	7.53%
Math Y6	4459	−1.31 [−1.72, −0.89]	55	6.6 × 10^−10^	11.48%
Math Y8	1603	−0.95 [−1.65, −0.26]	65	0.007	10.98%
M.A.	5343	−0.42 [−0.62, −0.23]	54	2.5 × 10^−5^	5.07%
Science Y6	4026	−0.52 [−0.68, −0.351	47	6.0 × 10^−10^	7.39%

*^a^Adjusted for maternal age, consumption of oily fish in pregnancy, smoking cigarettes mid pregnancy, binge drinking mid-pregnancy, breast feeding, visits to library, visits to places of interest, mother sings to child, mother reads to child, parenting score, child allowed objects for building, exposed to environmental tobacco smoke, child’s diet is poor (named ‘junk food diet’). ^b^Reduction in the adjusted beta coefficient for external locus of control compared with results in Table 3.*

### Explanation by School-Age Factors

For Analyses D we took account independently of the following variables: number of books owned by the child, whether children had been excluded from the child’s class, no. of children in the class receiving free school meals, teacher reports that the parents are very supportive toward the child’s learning, and the frequency with which the child does school homework. Unfortunately, the data provided by schools resulted in a reduction in numbers such that only 618 children could be considered in analyzing Mathematics in Year 8. Consequently, those relationships were omitted from Table 7. For all the remaining tests, these school-age learning related variables were responsible for between 31 and 47% of the poor achievements of the children of the external mothers.

**Table 7:** Reductions in the effect size of maternal external locus of control after taking account of primary school age factors^a^ as well as sex and parity, for each of the mental arithmetic, the maths comprehension, and the science scores: results of stepwise regression.

Test	*N*	Adjusted β [95%CI]	Reduction^b^ %	*P %*	*R*^2^ %
Math Y4	2553	−0.79 [−1.02, −0.56]	35	3.6 × 10^−11^	13.73
Math Y6	2090	−1.54 [−2.12, −0.96]	47	1.8 × 10^−7^	12.84
M.A.	2316	−0.64 [−0.93, −0.34]	31	2.6 × 10^−5^	7.04
Science Y6	2221	−0.54 [−0.75, −0.331	45	5.0 × 10^−7^	9.24

*^a^Adjusted for number of books owned by the child, whether children had been excluded from the child’s class, no. of children in the class receiving free school meals, teacher reports that the parents are very supportive toward the child’s learning, and the frequency with which the child does school homework. ^b^Reduction in the adjusted beta coefficient for external locus of control compared with results in Table 3.*

### Overall Explanation

The results of the full analyses are shown in Table 8. This illustrates the fact that the mothers’ external LOC remains significantly associated with poor achievement in these tests, but that around half of the difference in the scores achieved by children of parents who were internally oriented is related to behaviors and choices made by the parents in regard to lifestyle, parenting, and schooling.

**Table 8:** Reductions in the effect size of maternal external locus of control after taking account of prenatal, infancy, preschool and primary age factors^a^ as well as sex and parity, for each of the mental arithmetic, the maths comprehension, and the science scores.

Test	*N*	Adjusted β [95%CI]	Reduction^b^ %	*P*	*R*^2^ %
Math Y4	1806	−0.63 [−0.90, −0.35]	48	1.0 × 10^−5^	14.46
Math Y6	1953	−1.25 [−1.86, −0.64]	57	5.7 × 10^−5^	13.75
M.A.	2121	−0.48 [−0.79, −0.17]	49	0.003	7.71
Science Y6	1946	−0.49 [−0.71, −0.261	50	2.6 × 10^−5^	10.41

*^a^See text for list of factors offered to the analysis. ^b^Reduction in the adjusted beta coefficient for external locus of control compared with results in Table 3.*

## Discussion

### Overview of Results

We hypothesized and found that children whose mothers were more external : (a) have poorer educational achievements; and (b) that the relationships between the maternal LOC and the child’s mathematical and science abilities would be mediated through maternal lifestyle, parenting behaviors and attitudes to education. Children of mothers who are externally oriented scored significantly lower on tests of mathematics and scientific understanding than children of mothers who are internally controlled. At least half of the variance was associated with mothers’ parenting behaviors and lifestyles.

### Placing the Results in Context

The present study concentrated on assessments of mathematical and science reasoning which were designed specifically for the ALSPAC study and administered in schools. We used these data rather than data from national tests, because they assessed mathematical and scientific reasoning. Past research has shown that these maths tests (as assessed in school year 4), together with the child’s ability in mental arithmetic, make independent contributions to children’s achievement in mathematics in the national tests (Nunes et al., 2012). Additionally, the science reasoning test has been shown to be strongly predictive of the results from subsequent national science tests (Bryant et al., 2015).

This is not the first study using the ALSPAC cohort to have shown a cognitive association with maternal LOC. Previously, Golding et al. (2017a) found maternal external LOC to be associated with decreased ability in offspring IQ, as measured using the WISC, with children of externally oriented mothers having a disadvantage of approximately 7 IQ points at age 8. In that study we also investigated possible mechanistic explanations for the results by determining the extent to which three separate sets of factors known to be associated with the LOC orientation might explain the findings. We found (a) perinatal life-style exposures, (b) parenting attitudes and strategies and (c) socio-economic circumstances, largely explained the mechanism through which the externality of the mother may be associated with influencing the cognition of the child. In fact, perinatal life-style exposures and parenting attitudes and strategies explained about 50% of the difference found between the abilities of children of internal and external mothers.

### Limitations of the Study

We list our study limitations below.

(1) We did not allow for concurrent social circumstances as we wished to concentrate on factors that could be changed for mothers to become less external and more internal.
(2) The mental arithmetic scores used were confined to the children who attended the clinic held at the ALSPAC offices when the children were aged 8. As with all such attendances, there was a bias in that children whose mothers were externally oriented were less likely to attend. This bias must be borne in mind when interpreting the results.
(3) It is possible that there are other confounders that should have been taken into account, and which would have reduced the associations.
(4) To our knowledge there are no similar longitudinal datasets which could be used to determine whether these results can be repeated. Such repetition would currently provide additional support for our hypotheses.

### Strengths of the Study

(1) In comparison with studies on this topic, this sample size is numerically large.
(2) Although almost all longitudinal studies find attrition in attendance for measurements as their study population ages, in contrast, for the ALSPAC tests of maths and science reasoning, all children in a school class were included, and consequently bias related to the LOC of the study mother was reduced.
(3) The mothers’ LOC was measured in the first 6 months of pregnancy (90% by 28 weeks gestation), and can be considered a baseline, largely unaffected by the outcome of the pregnancy. The consistency of the LOC personality characteristic over time is shown by the correlations of the pregnancy measure with repeated measures of her LOC (*r* = 0.55 and 0.54) using the same questions obtained after 6 and 18 years respectively (Nowicki et al., 2018).

### Is the Relationship Causal?

We demonstrated significant associations between mothers’ LOC and their children’s mathematics and science reasoning tasks, but a key question concerns whether the findings imply causality. One set of results is consistent with such an assumption; factors we have shown elsewhere to explain about half of the original association with both maternal LOC and subsequent parenting behavior.

There are two ways in which the relationship between maternal LOC and the child’s scholastic abilities could be more convincingly shown to be causal – the first concerns the repetition of the findings in other communities, but more convincing would be the undertaking of randomized controlled trials comparing groups who had been encouraged to become more internal with those where no such intervention had occurred. Opportunities for such interventions could be based in secondary schools and/or provided to women attending antenatal classes.

## Conclusion

In this paper we have used tests of mathematical and scientific understanding since they illustrate fundamental abilities which will provide the basis for educational achievement in technical spheres in the future. We have concentrated the analysis on features of the mother that could be altered (in theory at least). It is also true that because LOC orientation is learned, it can be changed at any stage of life through learning. In the present study, we showed that children of an externally oriented mother are more likely to score poorly on tests concerning the understanding of fundamental mathematical and scientific concepts compared to children of an internally controlled mother, and, importantly, we found that about half of this is due to features of the behavior of the mothers. Programs to enable mothers to become more internally oriented may also help them use behaviors resulting in long-term benefit to the child’s educational achievements, but further research is needed to ensure that the relationship we have shown between parental LOC and child achievement results is causal.

## Author Contributions

JG and SN raised the funding; had the idea and wrote the first draft. TN and PB advised on the interpretation of the measures. SG and GE analyzed the data. YI-C contributed to the initial manuscript. All authors were subsequently involved in re-writing and editing.

## Conflict of Interest Statement

The authors declare that the research was conducted in the absence of any commercial or financial relationships that could be construed as a potential conflict of interest.
