# Supplementary material for: Maternal Prenatal External Locus of Control and Reduced Mathematical and Science Abilities in Their Offspring: A Longitudinal Birth Cohort Study
Source: Front Psychol. 2019 Feb 8;10:194. doi: 10.3389/fpsyg.2019.00194 (PMC6375836; doi:10.3389/fpsyg.2019.00194)
Supplement: Supplementary file 1 [file Table_1.DOCX]

**Supplementary Table 1**. Descriptive data for backgrounds of the children who did each

of the various tests

| Variable | All in  ALSPAC | Maths  Year 4 | Maths  Year 6 | Maths  Year 8 | Mental  arithmetic | Science |
| --- | --- | --- | --- | --- | --- | --- |
| N | 13,988 | 6,821 | 10,342 | 3,382 | 7,395 | 10,261 |
| *Sex of offspring* |  |  |  |  |  |  |
| Boy | 51.8% | 50.8% | 50.5% | 51.2% | 49.7% | 50.7% |
| Girl | 48.2% | 49.2% | 49.5% | 48.8% | 50.3% | 49.3% |
| *Maternal age* |  |  |  |  |  |  |
| <25 | 24.0% | 21.7% | 22.0% | 18.4% | 14.7% | 22.0% |
| 25-34 | 66.1% | 68.0% | 68.2% | 70.5% | 72.9% | 68.3% |
| 35+ | 9.9% | 10.3% | 9.8% | 11.1% | 12.4% | 9.7% |
| *Maternal education* |  |  |  |  |  |  |
| Low (< O-level) | 30.6% | 27.9% | 29.2% | 25.4% | 21.7% | 29.4% |
| Medium (O-level) | 34.3% | 34.1% | 35.4% | 35.4% | 34.9% | 35.6% |
| High (A-level +) | 35.1% | 38.0% | 35.2% | 39.2% | 43.4% | 35.0% |
| *Parity* |  |  |  |  |  |  |
| First born | 45.2% | 45.4% | 45.5% | 44.8% | 46.9% | 45.5% |
| Later born | 54.8% | 54.6% | 54.5% | 55.2% | 53.1% | 54.5% |
| *Social class** |  |  |  |  |  |  |
| Non-manual | 55.6% | 57.5% | 55.2% | 58.9% | 61.7% | 54.8% |
| Manual | 44.4% | 42.5% | 44.8% | 41.1% | 38.3% | 45.2% |
| *Mother smoked** |  |  |  |  |  |  |
| Yes | 19.7% | 18.2% | 18.8% | 16.2% | 13.6% | 18.6% |
| No | 80.3% | 81.8% | 81.2% | 83.8% | 86.4% | 81.4% |
| *Alcohol consumption** | |  |  |  |  |  |
| None | 50.6% | 49.6% | 50.9% | 50.5% | 47.8% | 51.2% |
| < once a week | 34.5% | 35.4% | 34.4% | 33.7% | 36.4% | 34.6% |
| ≥ once a week | 14.9% | 15.0% | 14.5% | 15.7% | 15.8% | 14.2% |

*Social class based on father’s occupation; smoking and alcohol exposure mid-pregnancy.

**Supplementary Table 2**. Descriptive data for the outcome measures

| **Outcome variable** | **Mean (SD)** | **Median** | **Range** |
| --- | --- | --- | --- |
|  |  |  |  |
| Maths comprehension Year 4 | 10.5 (3.2) | 11 | 0-17 |
|  |  |  |  |
| Maths comprehension Year 6 | 18.8 (7.1) | 19 | 0-35 |
|  |  |  |  |
| Maths comprehension Year 8 | 22.9 (7.4) | 24 | 0-35 |
|  |  |  |  |
| Mental arithmetic at age 8 | 14.8 (3.5) | 14 | 0-28 |
|  |  |  |  |
| Science Year 6 | 5.8 (2.6) | 6 | 0-10 |
|  |  |  |  |
